# Knockout of the epilepsy gene *Depdc5* in mice causes severe embryonic dysmorphology with hyperactivity of mTORC1 signalling

**DOI:** 10.1038/s41598-017-12574-2

**Published:** 2017-10-03

**Authors:** James Hughes, Ruby Dawson, Melinda Tea, Dale McAninch, Sandra Piltz, Dominique Jackson, Laura Stewart, Michael G. Ricos, Leanne M. Dibbens, Natasha L. Harvey, Paul Thomas

**Affiliations:** 1 School of Biological Sciences, University of Adelaide, Adelaide, SA AUS 5005 Australia; 2 Robinson Research Institute, University of Adelaide, Adelaide, SA AUS 5005 Australia; 3 Centre for Cancer Biology, University of South Australia and SA Pathology, Adelaide, SA AUS 5000 Australia; 4 University of South Australia, Epilepsy Research Program, School of Pharmacy and Medical Sciences, Adelaide, SA AUS 5000 Australia

## Abstract

*DEPDC5* mutations have recently been shown to cause epilepsy in humans. Evidence from *in vitro* studies has implicated DEPDC5 as a negative regulator of mTORC1 during amino acid insufficiency as part of the GATOR1 complex. To investigate the role of DEPDC5 *in vivo* we generated a null mouse model using targeted CRISPR mutagenesis. *Depdc5* homozygotes display severe phenotypic defects between 12.5-15.5 dpc, including hypotrophy, anaemia, oedema, and cranial dysmorphology as well as blood and lymphatic vascular defects. mTORC1 hyperactivity was observed in the brain of knockout embryos and in fibroblasts and neurospheres isolated from knockout embryos and cultured in nutrient deprived conditions. Heterozygous mice appeared to be normal and we found no evidence of increased susceptibility to seizures or tumorigenesis. Together, these data support mTORC1 hyperactivation as the likely pathogenic mechanism that underpins *DEPDC5* loss of function in humans and highlights the potential utility of mTORC1 inhibitors in the treatment of *DEPDC5*-associated epilepsy.

## Introduction

Epilepsy is a collection of neurological disorders characterised by unprovoked seizures. Focal epilepsy is the most common type of epilepsy and is caused by a range of genetic and environmental factors. In focal epilepsies seizures originate from a specific region of the brain as opposed to the generalised epilepsies where seizures originate from both hemispheres of the brain simultaneously. In 2013 we demonstrated that heterozygous *DEPDC5* mutations cause autosomal dominant focal epilepsies with variable expressivity and incomplete penetrance^1^. Atypical for genetic epilepsies, the locus of seizure origin is variable, even among affected members of the same family, and can include frontal, temporal, fronto-temporal, parietal and occipital regions of the human cortex. Since our initial observations were published, *DEPDC5* mutations have emerged as a major cause of inherited focal epilepsy, with mutations also reported in cases of autosomal dominant nocturnal frontal lobe epilepsy (ADNFLE), familial temporal lobe epilepsy (FTLE), benign epilepsy with centrotemporal spikes (BECTS) and other small families and individuals with focal epilepsy^2,3^. A number of *DEPDC5* mutations are frame shift or nonsense changes, indicating that they are likely to cause loss of function. Together these studies identify *DEPDC5* as an important new genetic cause of focal epilepsy.

*DEPDC5* encodes a 1604 amino acid protein that, with NPRL2 and NPRL3, forms the GTPase-activating-protein (GAP) Activity TOward Rags (GATOR1) complex 1^4^. Interestingly, we have shown that mutations of *NPRL2* and *NPRL*3 also cause focal epilepsy^4,5^. GATOR1 inhibits mTORC1 when amino acids are in short supply by functioning as a GTPase-activating protein (GAP) for the Rag subfamily of small GTPases that normally facilitate mTORC1 translocation to its active site at the lysosomal membrane. The importance of the GATOR1 complex in amino acid sensing by mTORC1 is highlighted by its exquisite conservation from yeast to humans where the analogous yeast protein complex SEACIT is composed of Npr2p, Npr3p and Iml1p^6^.

Mutations in GATOR1 genes give rise to patient phenotypes that overlap with Tuberous Sclerosis Complex (TSC), caused by mutations in *TSC1* and *TSC2*. These genes encode proteins that form the tuberous sclerosis complex that also acts to negatively regulate mTORC1 signalling. While GATOR1 mediates repression of mTORC1 during nutrient starvation conditions, the TSC protein complex inhibits mTORC1 activity during limited growth factor availability^7,8^. Mutations in GATOR1 complex genes and TSC genes are both associated with epilepsy, brain malformations, autistic features and intellectual disability. It is not yet clear how the pathobiological mechanisms leading to these phenotypes are linked.

To further investigate the function of DEPDC5 *in vivo*, we generated mice with loss of function alleles using CRISPR/Cas9 mutagenesis. We found that *Depdc5* null mice died during embryogenesis, exhibiting retarded growth, anaemia, eye, liver, cranial and vascular defects. mTORC1 hyperactivation was detected in embryonic brain lysates and nutrient starved neurospheres and MEFs. These data indicate that mTORC1 hyperactivation is a likely pathogenic mechanism that results from *DEPDC5* loss of function and points to the potential utility of mTORC1 inhibitors in the treatment of patients with *DEPDC5* mutations.

## Results

### Generation of *Depdc5* frameshift mutant mice using CRIPSR/Cas9 genome editing

**Figure 1:**
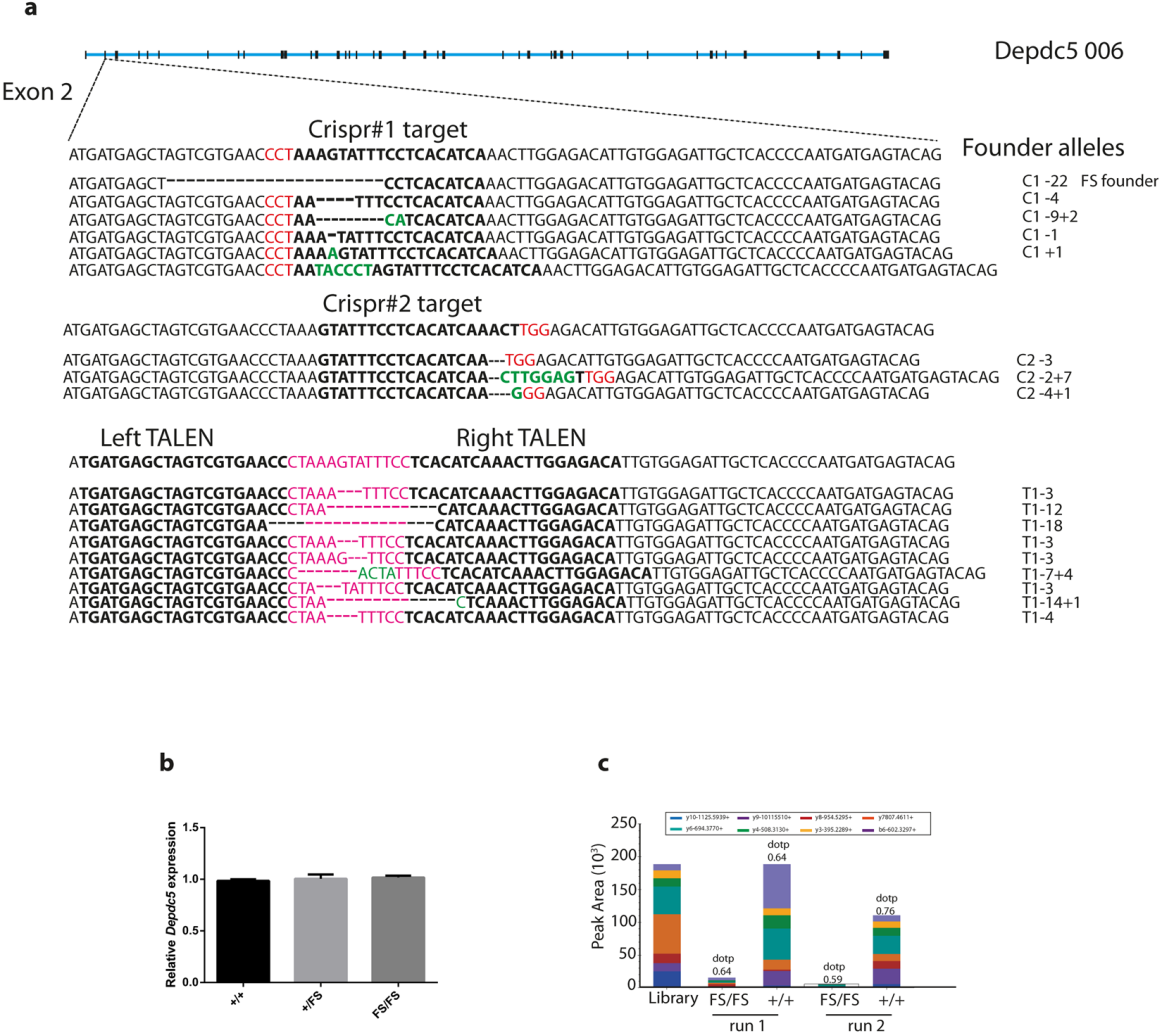
Generation of *Depdc5* null mice. (**a**) Exon 2 of mouse *Depdc5* was targeted with two separate CRISPR gRNAs or a pair of TALENs shown in bold (PAM shown in red, TALEN spacer shown in pink). (Frameshift Founder allele (FS) is depicted with deleted bases represented by dashes.) Founder alleles are depicted with deleted bases represented by dashes and inserted bases shown in green. (**b**) *Depdc5* expression was measured from cDNA generated from 3 *Depdc5* ^+/+^ and 3 *Depdc5* ^FS/FS^ 12.5 dpc brain RNA preparations and is expressed relative to beta actin expression. Two way comparisons were performed using unpaired two tailed Student’s t-tests, error bars represent SEM, n = 3. (**c**) Relative quantification of a DEPDC5 peptide ^195^AVNGFLADLFTK^206^ was performed using PRM on head lysate from one *Depdc5* ^FS/FS^ 12.5dpc embryo and one *Depdc5* ^+/+^ 12.5dpc embryo. Each colour represents the abundance of y- and b-ions of the protein of interest in comparison to the spectral library. dotp = dot product.

To generate *Depdc5* mutant mice we used TALEN and CRISPR/Cas9 genome editing technologies to induce double stranded breaks in exon 2 of *Depdc5*, with a view to creating frameshift mutations early in the open reading frame to disrupt protein function. Mouse zygotes were injected with either mRNA for a pair of TALENs or Cas9 mRNA and gRNA, and transferred to psuedopregnant recipients for development to term. Two separate CRISPR gRNAs (C1 and C2) and one TALEN pair (T1) were used. Twelve founder mice carrying a total of 18 mutant alleles were generated (Fig. 1a). Interestingly, founders from TALEN injections did not contain any WT alleles, while both CRISPR guides resulted in founders with a mixture of WT and mutant alleles, suggesting that the rate of mutagenesis was higher for the TALEN pair in this experiment. Many mosaic founders were generated from both CRISPR and TALEN injections, supporting observations previously reported of persistent endonuclease activity into the two cell stage and perhaps later^9–11^ Upon analysis of founder genotypes it was striking that all animals contained at least one WT allele or a small in frame deletion typically of 3 base pairs. The overrepresentation of these likely functional in frame deletion alleles combined with the high mutation frequency observed by us and others^9,12,13^ suggested that animals with frameshift mutations on both alleles did not survive to weaning.

Eight frameshift alleles were generated of which six were transmitted through the germline (Fig. 1a and Supplementary Fig. S1a). A 22 base pair deletion (c77_99del/L23fsX65) derived from CRISPR Founder 1 (Fig. 1a, Supplementary Fig. S1a) was selected for further analysis due to ease of genotyping (Supplementary Fig. S1b) and is referred to hereafter as frameshift (FS). The intercross of FS heterozygotes failed to generate *Depdc*5^FS/FS^ animals at weaning, further supporting the occurrence of FS homozygous embryonic lethality (Table 1, p < 0.0001). We anticipated that the frameshift in exon 2 adjacent to an intron/exon boundary may result in lower transcript levels due to nonsense-mediated decay, however we could detect no difference in transcript level between *Depdc5* ^+/+^ and *Depdc*5^FS/FS^ tissue (Fig. 1b). Using *Depdc*5^FS/FS^ and *Depdc5* ^+/+^ protein lysate we were unable to identify an antibody specific for DEPDC5, including a previously published^1^ antibody (D19 rabbit polyclonal antibody (Santa Cruz Biotechnology, sc-86116), we therefore confirmed the absence of DEPDC5 protein in *Depdc*5^FS/FS^ animals using the mass spectrometry technique of parallel reaction monitoring (PRM). PRM is a highly sensitive form of targeted mass spectrometry that allows the relative quantification of peptides. Figure 1c shows a representation of the relative quantitation of a transition for peptide ^195^AVNGFLADLFTK^206^ in comparison to the spectral library control. We noted some variability in the enrichment of y- and b-ions that we speculate is due to the low abundance of the peptide. Nevertheless these differences in enrichments are within acceptable tolerance. In addition an independent peptide (^1238^TFIYGFYFY^1247^) was detected by Selection Ion Monitoring (SIM) nano-LC-ESI-MS/MS in *Depcd5* ^+/+^ lysate but not in *Depdc*5^FS/FS^ lysate (Supplementary Fig. S2). Both peptides are downstream of the CRISPR target site in exon 2 and are encoded by exons 10–11 and 37 respectively, which are collectively present in all annotated mouse transcripts. Given the absence of these DEPDC5 peptides we conclude that FS constitutes a null allele.

**Table 1:** Chi squared test for postnatal offspring from *Depdc5* ^FS/+^ x *Depdc5* ^FS/+^ cross, (p < 0.0001).

	+/+	FS/+	FS/FS
Observed	37	57	0****
Expected	23.5	47	23.5

**Figure 2:**
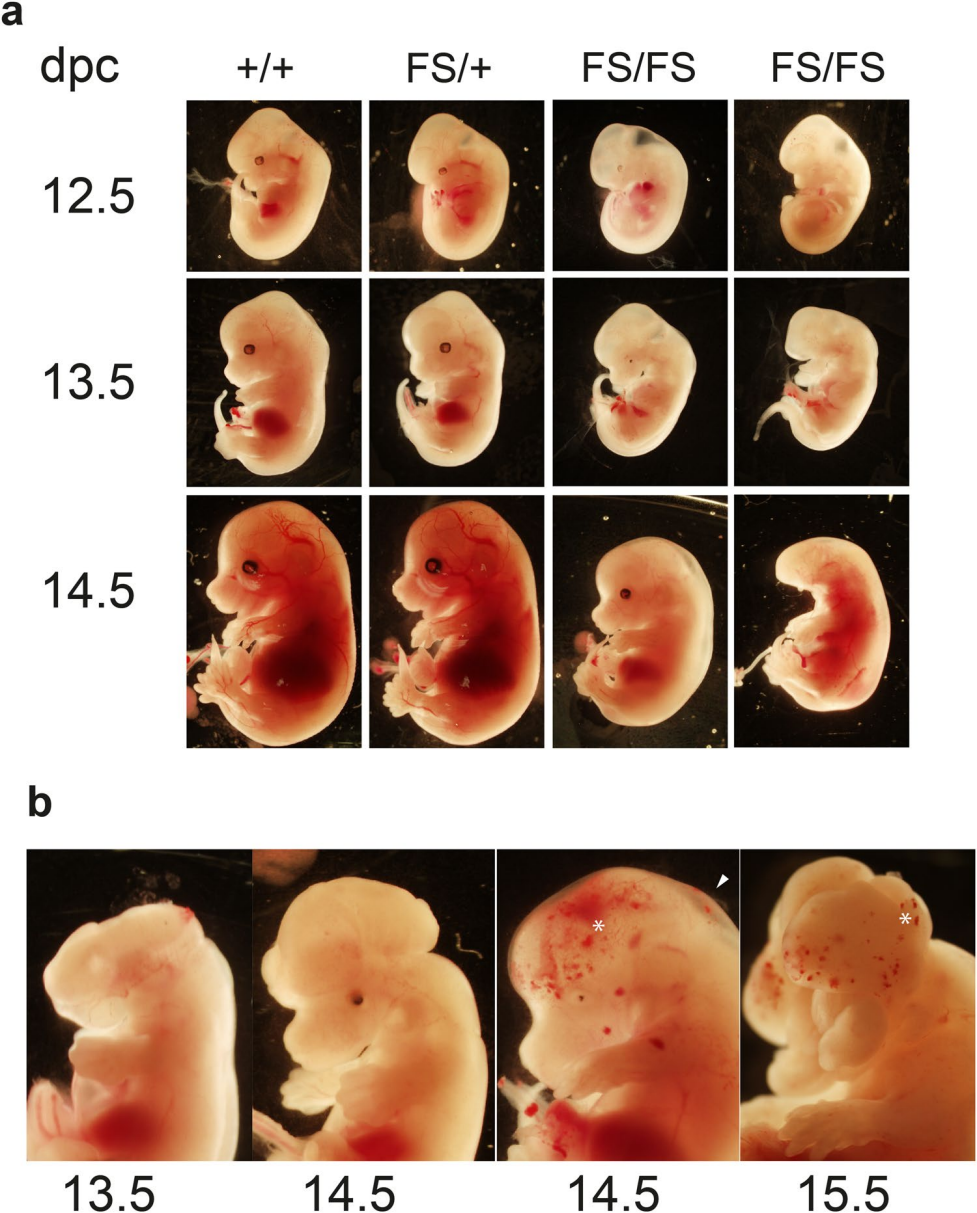
Gross anatomy of midgestational embryos. (**a**) Representative embryos for each genotype at 12.5, 13.5 and 14.5 dpc. Total numbers observed were; FS/FS, 12.5dpc = 6, 13.5dpc = 4, 14.5dpc = 8, FS/+, 12.5dpc = 16, 13.5dpc = 15, 14.5dpc = 29, +/+, 12.5dpc = 9, 13.5dpc = 9, 14.5dpc = 9. The phenotypic variability of *Depdc5* ^FS/FS^ embryos is represented with a mild and severe example at each time point. (**b**) Examples of cranial dysmorphology at 13.5, 14.5 and 15.5 dpc. Oedema (arrow head) and haemorrhaging (asterisk) is shown.

To further investigate the timing of phenotypic onset in FS mice, we harvested embryos from heterozygous crosses at various stages of gestation. Only viable embryos with a discernible heartbeat were included. *Depdc*5^FS/FS^ embryos at 9.5dpc were macroscopically normal, while from 10.5-15.5 dpc displayed a range of developmental abnormalities including reduced size, micropthalmia/anopthalmia and hepatic hypoplasia. At 10.5 dpc the most penetrant phenotypes in *Depdc*5^FS/FS^ embryos were reduced size (67%, Table 2) and micropthalmia/anopthalmia (44%, Table 2). Micropthalmia/anopthalmia and hepatic hypertrophy were highly penetrant by day 13.5dpc and at 14.5 dpc the majority of *Depdc*5^FS/FS^ embryos displayed overt cranial dysmorphology (88%, Fig. 2 and Table 2). We noted that the severity of abnormalities was also variable within each time point and present examples representative of each end of the spectrum for completeness (Fig. 2). The onset of severe phenotypes at midgestation indicates that DEPDC5 has an essential role during embryogenesis. This finding is consistent with our recently published expression data showing that *Depdc5* transcript is expressed throughout mouse development with a modest peak at 12.5dpc^5^. *Depdc*5^FS/FS^ embryos appear to be underrepresented when 12.5–17.5dpc embryo numbers were pooled (Table 3, Chi-square test, p = 0.051), suggesting variable lethality across this period of development rather than one single day which is likely due to the variability seen in severity of phenotypes.

**Table 2:** Phenotype frequencies of *Depdc*5^FS/+^ and *Depdc*5^FS/FS^ embryos.

Phenotype		10.5dpc	12.5dpc	13.5dpc	14.5dpc
Reduced embryonic size	FS/FS	6/9 = 67%	3/6 = 50%	3/4 = 75%	3/8 = 38%
	FS/+	4/7 = 23%	0/16 = 0%	0/15 = 0%	2/29 = 7%
	+/+	0/4 = 0%	0/9 = 0%	0/9 = 0%	1/9 = 11%
Micropthalmia/anopthalmia	FS/FS	4/9 = 44%	5/6 = 83%	4/4 = 100%	8/8 = 100%
	FS/+	0/17 = 0%	0/16 = 0%	1/15 = 7%	2/29 = 7%
	+/+	0/4 = 0%	0/9 = 0%	0/9 = 0%	0/9 = 0%
Hepatic hypoplasia	FS/FS	1/9 = 11%	3/6 = 50%	4/4 = 100%	5/8 = 63%
	FS/+	0/17 = 0%	0/16 = 0%	0/15 = 0%	2/29 = 7%
	+/+	0/4 = 0%	0/9 = 0%	0/9 = 0%	0/9 = 0%
Cranial dysmorphology	FS/FS	0/9 = 0%	0/6 = 0%	1/4 = 25%	7/8 = 88%
	FS/+	0/17 = 0%	0/16 = 0%	0/15 = 0%	0/29 = 0%
	+/+	0/4 = 0%	0/9 = 0%	0/9 = 0%	0/9 = 0%

**Table 3:** Numbers each genotype of viable embryos collected between 12.5–17.5dpc.

	+/+	FS/+	FS/FS
Observed	34	81	25
Expected	35	70	35

Chi squared test for viable 12.5–17.5dpc embryos from *Depdc5* ^FS/+^ x *Depdc5* ^FS/+^ cross, (p = 0.051).

**Figure 3:**
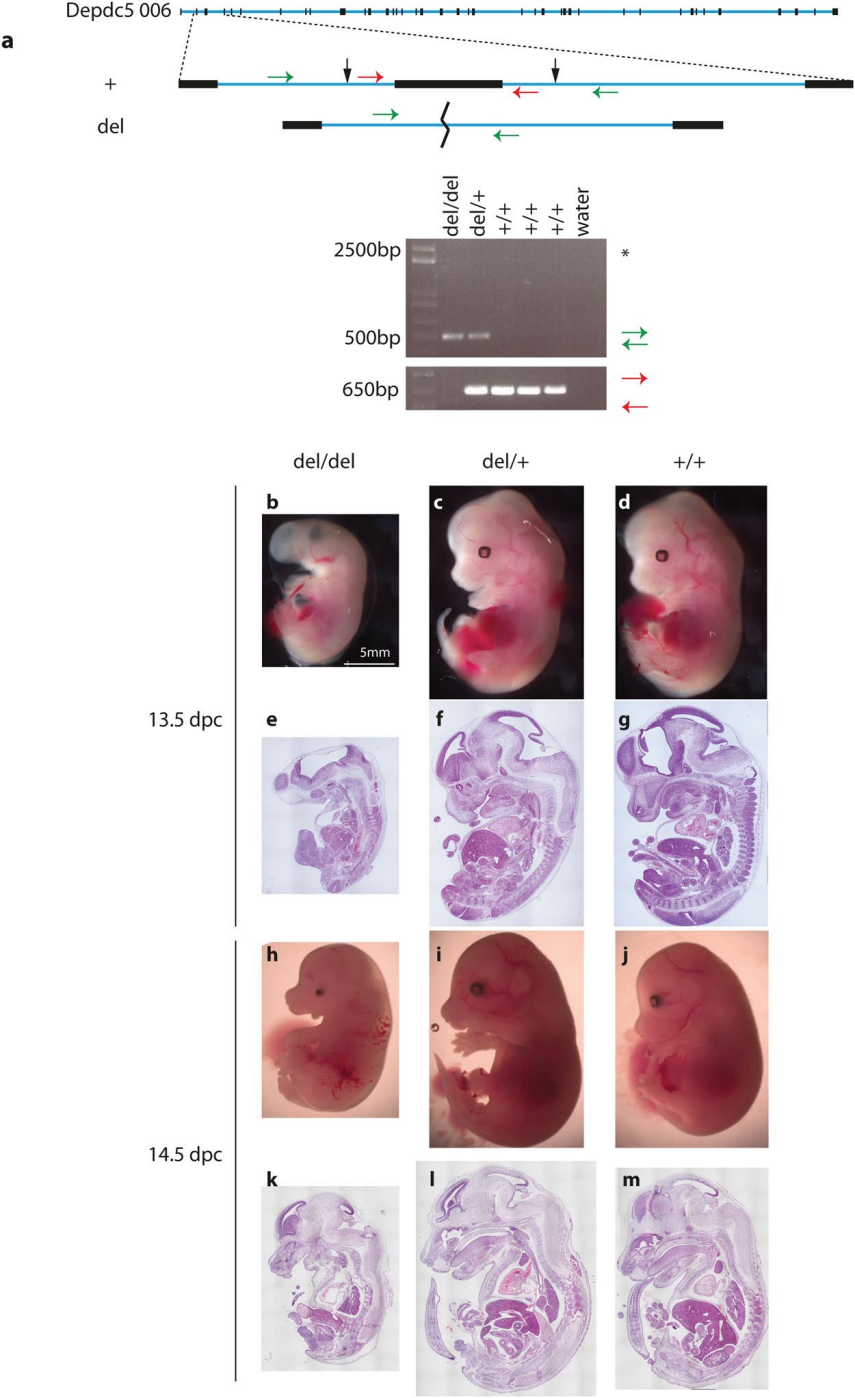
Independent *Depdc5* null mice phenocopy *Depdc5* ^FS/FS^ mice. (**a**) Two independent gRNA directed to intronic sequence flanking exon 2 (black arrows) lead to the deletion of exon 2 (del). PCR primers external to the deletion (green arrows) generate a 500 bp band after deletion. (*Note that amplification of the expected 2500 bp WT allele fails due to short extension time). PCR with primers internal to the deletion (red arrows), give a 650 bp band in WT allele only. Gross anatomy (**b–d**,**h–j**) and corresponding H&E (**e–g**, **k–m**) showing the phenotyope of del/del embryos.

CRISPR mutagenesis is known to induce off target mutations at a low frequency in a sequence specific manner dictated by the gRNA sequence^14,15^. To confirm that the observed embryonic lethality was due to mutation of *Depdc5* and not an off-target event, we generated an independent mutant line using an alternate strategy in which two gRNAs were directed against intronic sequence flanking exon 2 (Fig. 3a). Transmitting founders containing the intended frameshifting deletion of exon 2 (del) were identified. Morphological and histological analysis of del/del embryos at 13.5 and 14.5 dpc revealed identical abnormalities to *Depdc*5^FS/FS^ embryos including severe cranial dysmorphology, hepatic hypoplasia, reduced overall size and micropthalmia/anopthalmia and oedema (Fig. 3b–m). Taken together, these data confirm that the observed developmental defects result from *Depdc5* loss of function.

### Oedema in *Depdc5* mutants is associated with defects in cardiovascular development

**Figure 4:**
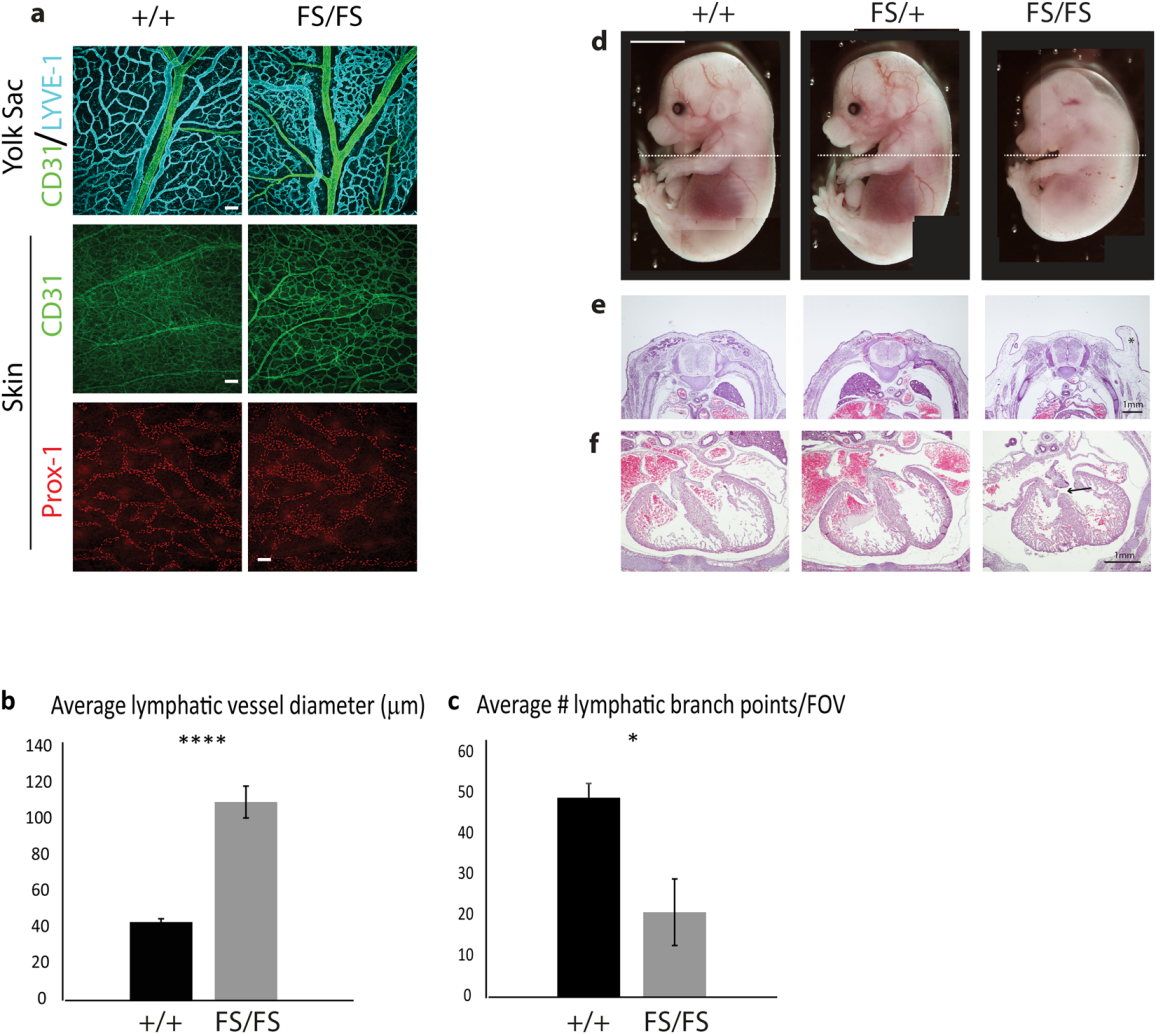
*Depdc5* ^FS/FS^ embryos exhibit defects in blood and lymphatic vascular patterning. (**a**) Yolk sacs of 14.5 dpc wild-type and *Depdc5* ^FS/FS^ embryos stained with CD31 (green) and LYVE-1 (cyan) revealed tortuous blood vessels in *Depdc5* ^FS/FS^ embryos. Skin of *Depdc5* ^FS/FS^ embryos exhibit reduced blood vessel density. Dermal lymphatic vessels (Prox1-positive, red) of 14.5 dpc *Depdc5* ^FS/FS^ embryos were significantly larger in calibre (****p < 0.0001) (**a**,**b**) and less branched (*p = 0.04953) (**a**, **c**) than wild-type counterparts. Two way comparisons were performed using Mann Whitney U test, error bars represent SEM, n = 3, FOV = field of view. White scale bars represent 100μm. (**d**) Gross anatomy of 15.5dpc embryos showing the plane of sectioning for **e** and **f**. Hematoxylin and Eosin stained sections (**e** and **f**) showing oedema (**e**, asterisk) and ventricular septal defect in *Depdc5* ^FS/FS^ embryos (**f**, arrow).

At 14.5 dpc *Depdc*5^FS/FS^ embryos were observed with oedema around the dorsal cranium, trunk and heart as well as discrete haemorrhagic blood pools in the skin (Figs 2b and 3). As these phenotypes are often associated with blood and lymphatic vascular development, we reasoned that such developmental defects could contribute to the embryonic lethality in mutant embryos. To further investigate this phenotype, we performed morphological and morphometric analysis of mutant tissue derived from viable embryos immunostained for markers of the blood (CD31) and lymphatic (PROX1, LYVE-1) vasculature. Analysis of yolk sacs from 14.5 dpc *Depdc*5^FS/FS^ embryos revealed tortuous blood vessels. The skin of 14.5 dpc *Depdc*5^FS/FS^ embryos exhibited aberrant blood vascular patterning, reduced lymphatic vessel branching and increased lymphatic vessel diameter (Fig. 4a–c). The jugular lymph sacs of 14.5 dpc *Depdc*5^FS/FS^ embryos were also enlarged (data not shown). Histological examination of 15.5 dpc *Depdc*5^FS/FS^ embryos confirmed the oedema observed by gross examination and revealed ventral septal dysplasia in the heart (Fig. 4d–f). These data reveal that *Depdc5* is required both for cardiac development and for blood and lymphatic vascular development and suggest that abnormalities of the cardiovascular system may be the primary cause of *Depdc*5^FS/FS^ embryonic lethality.

### Abnormal mTORC1 signalling in *Depdc5* mutants

**Figure 5:**
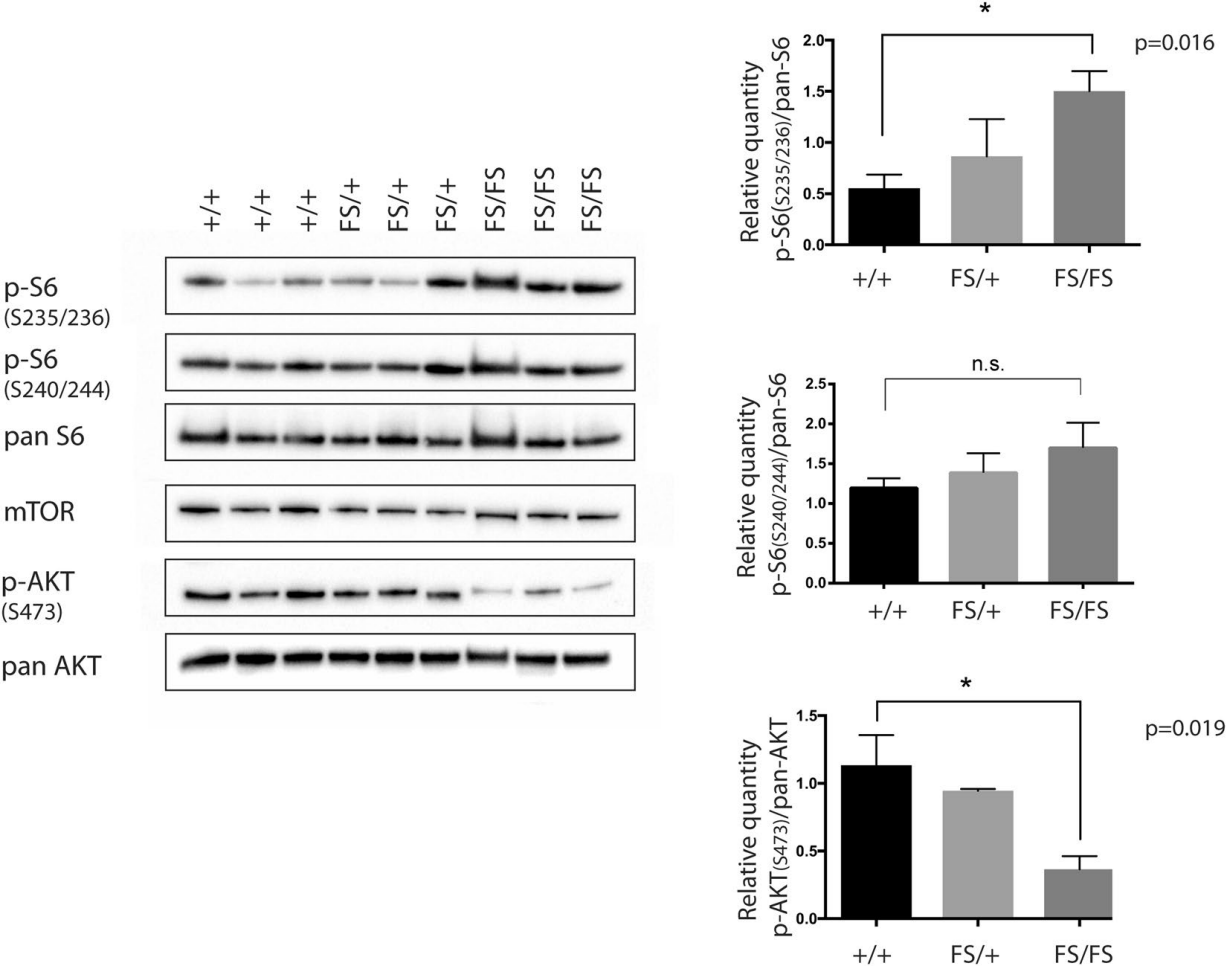
mTORC1 signalling is upregulated in *Depdc5* ^FS/FS^ embryos. Three independent 13.5 dpc brain lysates from each genotype were used. Densitometric quantitation was performed and analysed with unpaired two-tailed Student’s t-tests, error bars represent SEM, n = 3, *p < 0.05, n.s. = not significant. Separate blots were used for each antibody using the same experimental samples and were processed in parallel. The images shown are cropped. Full-length blots and original images are shown in Supplementary Fig. S3.

Published *in vitro* studies indicate that DEPDC5 functions as a negative regulator of mTORC1^4^. To investigate the impact of *Depdc5* loss-of-function on mTORC1 signalling *in vivo*, we performed Western blot analysis of 13.5 dpc embryonic brain, a tissue that expresses *Depdc5* and is severely affected by mutation of *DEPDC5* in patients. All embryos were viable at the time of collection. Markers of mTORC1 activity (Phosphorylated-S6-Ribosomal-protein (p-S6-S235/236 and p-S6-S240/244) and Phosphorylated-Ribosomal-protein-S6-kinase-beta-1 (p-p70S6k-T389) were significantly elevated in *Depdc*5^FS/FS^ samples, but not *Depdc5* ^FS/+^ samples, compared to *Depdc5* ^+/+^ controls (Fig. 5, full length blots are shown in Supplementary Fig. S3). We noted however that the level of hyperactivation was variable. This variability did not correlate with morphological severity but given that 13.5 dpc *Depdc*5^FS/FS^ embryos often exhibit profound developmental abnormalities (Fig. 2), we speculated that the direct effect of DEPDC5 loss of function could be masked in some samples due to secondary defects relating to embryo malformation and cell death. To address this we repeated our analysis on whole 9.5 dpc embryos, where gross morphological differences are not evident (data not shown). mTORC1 hyperactivation was also observed in *Depdc*5^FS/FS^ embryos at this stage with subtle upregulation of p-S6-S235/236 and more pronounced upregulation of p-p70S6k-T389 (Supplementary Fig. S4, full length blots are shown in Supplementary Fig. S5), suggesting that the increased mTORC1 activation is due to the loss of DEPDC5 function.

**Figure 6:**
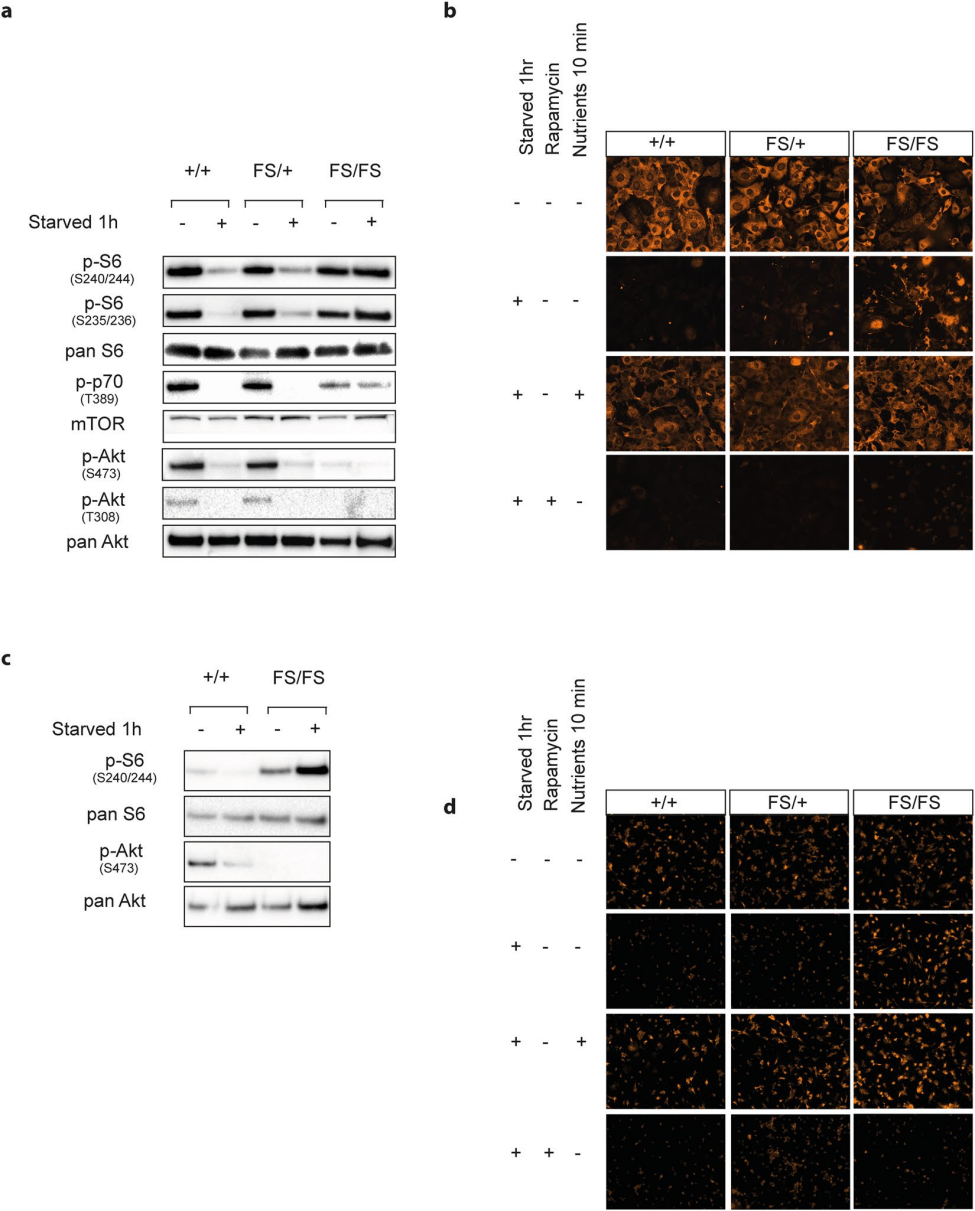
mTORC1 pathway upregulation following nutrient starvation in *Depdc5* ^FS/FS^ MEFs and neural progenitors. Immortalised MEFs (**a**,**b**) and differentiated neurospheres (**c**,**d**) were starved of nutrients for 1 hour before being collected for immunoblotting (**a**,**c**) or immunocytochemistry with anti-phospho-S6-S240/244 (**b**,**d**). *Depdc5* ^FS/FS^ MEFs fail to downregulate markers of mTORC1 activation after starvation (**a**,**b**). Specificity of p-S6-S240/244 downregulation in response to starvation is demonstrated by rapid upregulation following 10 minute return to nutrient replete basal media (nutrients 10 min). Rapamycin (20 nM) ablated all p-S6-S240/244 staining in both MEFs (**b**) and neural progenitors (**d**). Basal AKT activity is lower in *Depdc5* ^FS/FS^ MEFs and neuroprogenitors (starved minus lane *Depdc5* ^FS/FS^ vs *Depdc5* ^+/+^, **a**,**c**). Separate blots were used for each antibody using the same experimental samples and were processed in parallel. The images shown are cropped. Full-length blots or original images are shown in Supplementary Figures S6–7.

To assess the responsiveness of *Depdc5* null cells to amino acid starvation, we generated mouse embryonic fibroblasts (MEFs) and neurospheres from *Depdc*5^FS/FS^, *Depdc5* ^FS/+^ and *Depdc5* ^+/+^ animals and assessed mTORC1 activity as described above. Under basal, nutrient replete, conditions we did not detect any difference in mTORC1 downstream markers (p-S6-240/244, p-S6-240/244, p-p70-T389) in either MEFs or neuroprogenitors. However, when starved of amino acids and serum for 1 hour we observed increased mTORC1 signalling, as indicated by elevated levels of p-S6-240/244 in *Depdc*5^FS/FS^ cells but not *Depdc5* ^FS/+^ or *Depdc5* ^+/+^ cells (Fig. 6a–d, full length blots are shown in Supplementary Figures S6–7). The direct involvement of mTORC1 was confirmed by inclusion of rapamycin, an mTORC1 inhibitor, which supressed the mTORC1 hyperactivity in *Depdc*5^FS/FS^ cells (Fig. 6b,d). Together this data establishes that mTORC1 hyperactivation is observed in *Depdc*5^FS/FS^ tissues both *in vivo* and *in vitro* and is consistent with the described role of DEPDC5 as an inhibitor of mTORC1 under low amino acid conditions^4^.

To further explore the impact of DEPDC5 mutation on mTORC1 signalling, we assessed levels of phosphorylated-AKT (p-AKT-S473). This upstream component of the mTORC1 pathway is stimulated in response to insulin signalling and can also receive feedback inhibition from p-S6 in response to chronic mTORC1 hyperactivation^16^. Embryonic *Depdc*5^FS/FS^ lysates showed significant downregulation of p-AKT-S473 consistent with feedback inhibition downstream of mTORC1 hyperactivation (Fig. 5). Interestingly, p-AKT-S473 downregulation was more consistent than mTORC1 downstream marker upregulation, such that even those *Depdc*5^FS/FS^ lysates that did not exhibit pS6 upregulation still showed at least modest p-AKT downregulation (Supplementary Fig. S4). Similarly, in MEF and neurosphere culture, p-AKT-S473 and p-AKT-T308 downregulation was observed under nutrient replete conditions despite no apparent p-S6 upregulation (Fig. 6a,c).

### Seizure and tumour susceptibility in *Depdc5*^FS/+^ mice

**Figure 7:**
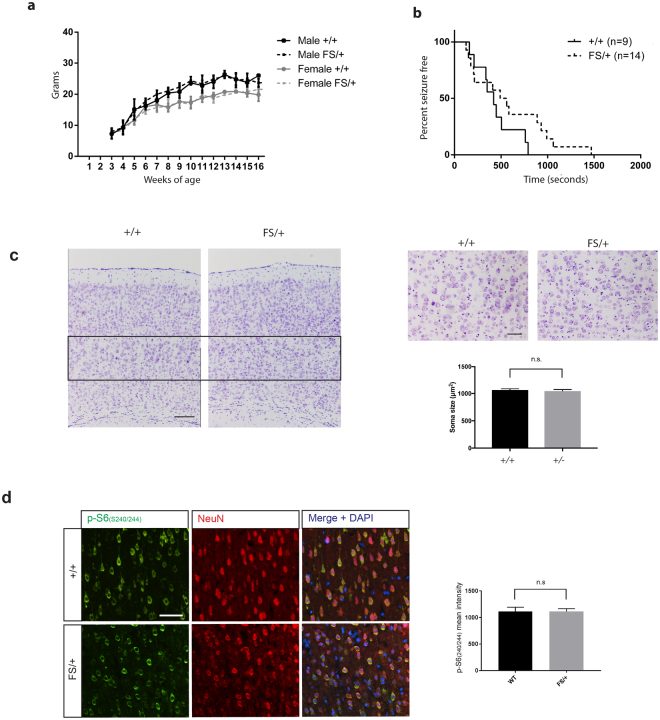
Heterozygous mice appear healthy, do not have overt seizures, have normal levels of p-S6-S240/244 in *Depdc5* ^FS/+^ adult brains and do not show cortical abnormalities. Mouse weights measured weekly from 3-16 weeks (**a**) were not different between *Depdc5* ^+/+^ and *Depdc5* ^FS/+^ for both sexes, error bars represent SD. (**b**) Latency to tonic clonic seizure following PTZ administration was not significantly different (p = 0.17) between 9 *Depdc5* ^+/+^ and 14 *Depdc5* ^FS/+^ 4-6 month old male mice as determined using a Mantel-Cox test. No mice were seizure-free at the end of the assay. (**c**) Representative Nissl-stained 12-24 week old mouse cortical sections. Three independent *Depdc5* mutant mouse strains were examined (*Depdc5* ^+/+^ n = 3, *Depdc5* ^FS/+^ n = 3). Left images have cortical layer V-VI boundary in black box which is enlarged in the right higher magnification images. Scale bars 250μm and 100μm respectively. Quantification of neuronal soma size in layer V of the cortex (n = 3 per genotype with 30 cells measured per animal). Two tailed Student’s t-test was used, error bars represent SEM. (**d**) 4–6 weeks old cortical brain slices were stained for p-S6-S240/244 (green) and NeuN (red) (n = 3, *Depdc5* ^+/+^, n = 3, *Depdc5* ^FS/+^, n = 3) White scale bar represents 50μm. Mean intensity of p-S6-S240/244 bright cells per field of view was not significantly different (n.s. p = 0.9990) between *Depdc5* ^+/+^ and *Depdc5* ^FS/+^ brains. (*Depdc5* ^+/+^, n = 3, *Depdc5* ^FS/+^, n = 3, at least 30 cells measured per animal across 6 images). Student’s t-tests were used, error bars represent SEM.

*Depdc5* ^FS/+^ mice, which may best reflect patients heterozygous for a *DEPDC5* mutation, exhibited normal growth and fertility (Fig. 7a). Given that humans with heterozygous mutations often develop epilepsy, we assessed the propensity of *Depdc5* ^FS/+^ mice to exhibit spontaneous seizures under normal conditions and through the administration of the proconvulsant Pentylenetetrazol (PTZ)^17^. No overt spontaneous seizures were detected during 30 minute daily observations of 8 female and 8 male heterozygous mice from weaning to 12 weeks under standard husbandry conditions. Nocturnal observation (when mice are most active) using Infrared (IR) recordings also failed to reveal overt spontaneous seizures in a cohort of 8 *Depdc5* ^FS/+^ and 6 *Depdc5* ^+/+^ mice. No significant difference in the PTZ-induced latency to tonic-clonic seizure was detected between a cohort of 4-6 month old 9 *Depdc5* ^+/+^ and 14 *Depdc5* ^FS/+^male mice (Fig. 7b). To investigate whether *Depdc5* heterozygous mice exhibit pathological features associated with some *DEPDC5* mutations in humans or other mTORC1 related pathologies such as giant or balloon cells^18–22^, we examined adult brains for cortical malformations and mTORC1 hyperactivation. Large p-S6-S240/244 bright cells have been reported in Depdc5 heterozygous rat brains^23^. Nissl-stained cells in layer V of the cortex were no larger in heterozygotes in comparison to wild type mice and histological analysis revealed no evidence of cortical malformations (Fig. 7c). Quantitative immunoanalysis for the mTORC1 hyperactivation marker p-S6-S240/244 in the cortex also did not reveal any significant difference between *Depdc5* ^FS/+^ and *Depdc5* ^+/+^ mice (Fig. 7d). Given that other mice with mTORC1 repressor gene knockouts, such as *TSC1 a*nd *TSC2*, show these phenotypes at comparable embryonic stages, we extended our analysis to older animals, where TSC heterozygotes show increased tumour incidence in the kidney and at other sites^24,25^. This would also be consistent with the observed role of the GATOR1 complex protein, NPRL2, as a tumour suppressor^26,27^. To investigate this we performed a phenotypic screen on two 12 month old and two 18 month old *Depdc5* ^FS/+^ mice but did not observe any unusual tissue pathology upon histological examination of 21 tissues and organs (data not shown).

## Discussion

Heterozygous mutation of *DEPDC5* is emerging as a relatively frequent cause of familial focal epilepsy. Focal epilepsy is the most common form of epilepsy in humans. *In vitro* functional studies indicate that DEPDC5 is an inhibitor of mTORC1 signalling, suggesting that *DEPDC5*-associated epilepsy may result from aberrant mTORC1 signalling, as is the case in tuberous sclerosis patients who often have epilepsy. To investigate the phenotypic consequences of *Depdc5* mutation *in vivo*, we generated *Depdc5* mutant mice using CRISPR/Cas9 and TALEN mutagenesis. Consistent with other studies utilizing these technologies to generate animal models, mutations were readily generated, highlighting the efficacy of zygotic genome modification for the generation of mouse models^9,12,28^. *Depdc*5^FS/FS^ animals die at mid to late gestation, with a range of phenotypes including cranial dysmorphology, general hypoplasia, micropthalmia/anopthalmia, oedema and haemorrhage. These phenotypes are also present in null embryos for the other GATOR1 complex members (*Nprl2* and *Nprl3*) as well as TSC1 and TSC2 and *Depdc5* null rats^23,29–32^, implicating mTORC1 hyperactivation in the observed lethality. We observed hyperactivation of mTORC1 in *Depdc*5^FS/FS^ embryos at midgestation and demonstrated that *Depdc*5^FS/FS^ mutant MEFs and neurospheres respond abnormally to nutrient starvation by retaining aberrantly high mTORC1 activity. Preliminary experiments were conducted attempting to rescue these embryonic phenotypes with the mTOR inhibitor rapamycin but were not conclusive and therefore further experimentation will be required to address this point. Together, our data demonstrate that DEPDC5 is essential for embryonic development and strongly suggest that aberrant mTORC1 activity is central to the mutant phenotype.

The phenotype of *Depdc*5^FS/FS^ embryos was variable, particularly with respect to cranial development. Some embryos were only mildly affected with no overt loss of tissue organisation while others had severe cranial atrophy with unrecognisable tissue organisation, including a loss of lateral ventricles, eyes and jaw structures, while retaining more normal trunk structures. We speculate that this variability may be secondary to an earlier embryonic defect that may represent the primary cause of lethality. Although mTORC1 signalling is likely to be exquisitely regulated in all tissues, a good candidate for cause of death in *Depdc*5^FS/FS^ embryos is defective cardiovascular development. Quantitative analysis of the developing blood vasculature and lymphatic vasculature in *Depdc5* null embryos revealed significant abnormities that likely underpinned the oedema and haemorrhaging that were observed. Notably, similar oedema, haemorrhaging and embryonic lethality have been reported in embryos with specific deletion of TSC in the developing vasculature^33^. Thus it appears likely that defective cardiovascular development is a major contributor to the lethality of *Depdc*5^FS/FS^ embryos.

DEPDC5 and TSC respond to different inputs into the mTORC1 pathway; amino acids and growth factors, respectively. We might therefore expect the phenotype of *Depdc5* and TSC null mice to reflect the abundance of these inputs during embryogenesis, such that defects should only be seen at times or in tissues where these inputs are limiting. The striking similarity of the *Depdc5* and TSC mutant phenotypes therefore suggests that both sets of stimuli are limiting during embryogenesis, which constitutes a period of very rapid cell growth and proliferation. The phenotypic similarity may also reflect a level of crosstalk between these two arms of the mTORC1 pathway such that perturbation of one arm results in defective sensing by the other arm. In support of this idea, it has been reported that TSC is able to sense amino acid deprivation via mTORC1 recruitment to the lysosomal membrane^34,35^. In the absence of TSC, mTORC1 lingers longer at the lysosomal membrane in low amino acid conditions, causing aberrant activity. It is possible that a similar mechanism may allow DEPDC5 to sense TSC inputs and if so, in the absence of DEPDC5, TSC would have reduced ability to switch off mTORC1 during low growth factor input. Further investigations are required to address these possibilities.

Given that DEPDC5 is ubiquitously expressed and that mTORC1 signalling is thought to be required in every cell of the developing embryo and adult, it is intriguing that humans with heterozygous *DEPDC5* mutations only develop an overt pathology within the CNS. This contrasts with TSC patients who exhibit lesions in multiple organs and display a diverse range of associated pathologies in addition to those that overlap DEPDC5-related phenotypes. Of note, many *Depdc*5^FS/FS^ embryos displayed severe cranial defects (Fig. 2), with several embryos showing more dramatic loss of head than trunk tissue. It is not clear why *Depdc5* loss of function in mice results in such severe cranial defects given its ubiquitous expression. It suggests that DEPDC5 and GATOR1 play more prominent role in brain development, which may help explain why epilepsy is the predominant phenotype reported in humans with *DEPDC5* mutations.

**Figure 8:**
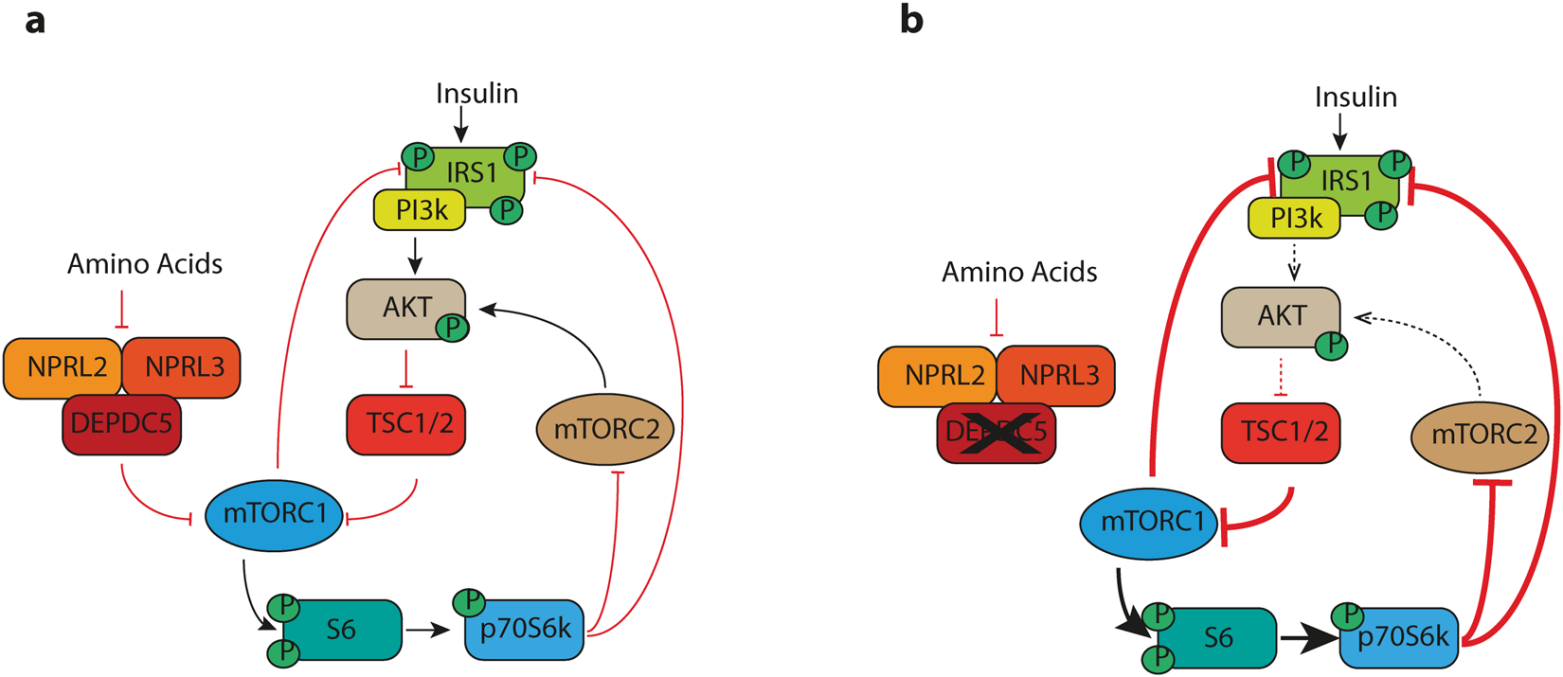
Model of signalling disruptions in *Depdc5* ^FS/FS^ mice. (**a**) DEPDC5 inhibition of mTORC1 is overcome by the presence of amino acids, while TSC1 inhibition of mTORC1 is overcome by growth factor signalling including insulin. Negative feedback loops exist to limit mTORC1 stimulation in times of chronic insulin stimulation. (**b**) Genetic ablation of *Depdc5* disrupts the GATOR1 complex and prevents the mTORC1 pathway from sensing amino acid levels. The consequent aberrant mTORC1 activity yields abnormally high phospho-S6 and phospho-p70S6k levels that subsequently provide negative feedback to reduce the levels of phospho-AKT. We predict that this may promote inhibition of mTORC1 by TSC1/2 and establish a tension with mTORC1 upregulation by DEPDC5 deletion.

In addition to mTORC1 deregulation, we detected consistent AKT hypophosphorylation in *Depdc*5^FS/FS^ tissues. The most parsimonious explanation for this finding is that it occurs via a negative feedback from phosphorylated S6 kinase to AKT. This well- established negative feedback loop has been reported to limit mTORC1 hyperactivation in situations of constitutive mTORC1 stimulation such as chronic insulin exposure, excess nutrients or genetic ablation of TSC^16^. This provides an interesting point of difference between TSC and *Depdc5* null mice, such that in *Depdc5* null tissues this feedback may have some ability to counteract mTORC1 hyperactivation, while in TSC null tissues AKT will be unable to influence mTORC1 due to the absence of the intervening TSC complex. This may result in more limited mTORC1 hyperactivation in *Depdc*5^FS/FS^ embryos than in TSC null animals. This difference in mTORC1 pathway regulation may also help explain the milder presentation of *DEPDC5* related disorders relative to TSC associated disease^18^ (Fig. 8).

Recently published *Depdc5* KO rats^23^ display many similarities with the mice reported here, including KO lethality, general growth retardation and mTORC1 hyperactivation, but important differences are also seen. Most notably, we saw no evidence of pathology in heterozygous mouse brains, while in rats, cortical lamination was disrupted and enlarged balloon-like and cytomegalic neurons were observed throughout the cortex and particularly in layers IV-V. This preponderance of abnormally large neurons was a surprising finding given that other murine heterozygotes lacking mTORC1 regulators (such as TSC) have not reported anything similar^24,25,29,36,37^. Indeed, the potential differences in gene expression, epigenome, rates of somatic mutations or neurogenic rate between the two species can lead to distinct phenotypes, especially for neurological diseases^38^. Furthermore, possible differences in the GATOR1/mTOR pathway (Fig. 8) may cause differences in sensitivity to loss of mTOR inhibition which could also contribute to species-specific effects. Recently it has been shown that random mono-allelic expression of *Depdc5* occurs in mouse brains^39^ which could also represent an important point of difference in rats versus mice. Generation and analysis of additional rat mTORopathy models, particularly those already modelled in mice, should help uncover the aetiology of species differences. In any event, the lack of seizures observed in both heterozygous mice and rats suggests that *DEPDC5* haploinsufficiency *per se* is not sufficient to cause epilepsy.

Absence of heterozygous pathology in mice is consistent with TSC mutant mice in which adult heterozygotes also fail to replicate clinical features associated with the human disease. Of note a recent publication reported seizures in TSC1 heterozygous mice that were subjected to conscious EEG recordings and were between 9–18 days old^40^. It will be interesting to perform similar further analyses on *Depdc5* mice at similar ages to see whether developmentally restricted seizures are also present. Indeed, EEG could detect rare or subclinical seizures that may be missed in behavioural observations/monitoring. Tellingly, mice with targeted deletion of both copies of *TSC1* or *TSC2* in the brain or specific brain regions are able to replicate many hallmarks of the human disease including the presence of tubers, enlarged p-S6 bright neurons and seizures^24,25,29,36,37,41–43^. Together with findings of Loss of Heterozygosity (LOH) in some human tubers, these observations have been used to support a second hit model underlying pathogenesis^44–46^. A recent report describing a somatic “second hit” mutation in *DEPDC5* in an individual with focal epilepsy and cortical dysplasia suggests that GATOR1 and TSC-associated epilepsies may share this underlying genetic mechanism^21^. It will now be important to produce a mouse model with targeted deletion of *Depdc5* in a discrete brain region to test this hypothesis.

In conclusion, we report that *Depdc5* null mice die at midgestation with mTORC1 hyperactivation and AKT downregulation. Heterozygous animals appear normal at all stages examined. Together with the remarkable similarity of *Depdc5* and *TSC* null mouse phenotypes our findings support the hypothesis that mTORC1 deregulation is likely to underpin *DEPDC5* related epilepsy and raises the possibility that mTORC1 inhibition may be a viable therapeutic option in patients with *DEPDC5* mutations as is being investigated for TSC patients^47^ (reviewed in^48^).

## Materials and Methods

### Generation and genotyping of mutant mice

Gene editing reagents were designed and purchased from ToolGen (South Korea). A TALEN pair was designed to target exon 2 of *Depdc5* (5′-TGATGAGCTAGTCGTGAACC-3′) (5′-TCACATCAAACTTGGAGACA-3′). Two independent CRISPR guides were designed adjacent to the TALEN spacer region targeting 5′-TGATGTGAGGAAATACTTT-3′and 5′-GTATTTCCTCACATCAAACT-3′. TALEN mRNA pairs (40 ng/μL each) or Cas9 mRNA (50 ng/μL) with crRNA (33 ng/μL) and tracRNA (67 ng/μL) were injected into C57BL/6 N zygotes, transferred to psuedopregnant recipients and allowed to develop to term. Founder pups were screened for indels by PCR amplification across the targeted region (F-5′-ATACCAACATGATGGGGTGCC-*3*′, R-5′-GATCAGCAGGGTGCCTACTTT-3′) followed by digestion with T7 endonuclease (NEB). PCR products from indel-carrying founders were Sanger sequenced to identify specific mutations.

Routine genotyping was performed by polyacrylamide gel electrophoresis in which PCR products generated with the above primers spanning the target region were separated on 15% polyacrylamide TBA gels. Homoduplexes run at the expected sizes, while heteroduplexes had retarded electromobility.

All animal work was conducted following approval by The University of Adelaide Animal Ethics Committee (approval number S-2013-186) in accordance with the Australian code for the care and use of animals for scientific purposes.

### Histological analyses

Embryos for histological examination were fixed in 4% PFA, embedded in paraffin, cut at 6μm and stained with Mayer’s hematoxalin and Eosin using standard protocols. Nissl (C5042) staining was performed using standard protocols on perfused brains of three mice from three independent *Depdc5* mutant mouse strains. All mouse lines targeted the same exon and generated a frameshift mutation (−22bp, −4bp as shown in Fig. 1 and −2 kb as shown in Fig. 3). Images were captured and analysed using Nikon Eclipse Ti microscope and Nikon Digital Sight DS-Qi1 camera.

### Real time PCR

RNA was extracted from 12.5 dpc brains by using Trizol (Thermo Fisher) and purified further on an RNeasy mini column (Qiagen). Reverse transcription was performed on 1 μg of RNA using a High Capacity RNA to cDNA synthesis kit (Life Technologies). Real time PCR was performed on a Step One Plus thermocycler (Life Technologies) using Fast Sybr green master mix (Life Technologies) according to manufacturer’s instructions on a two-step programme. Primers and product sizes were as follows; *Depdc5* (109 bp) F-5′-TGGGGACAAACCCCGTGCAG-3′ R-5′-CATGCGGTCTGAGCGGTGGC-3′, *β-actin* (89 bp) F-5′-CTGCCTGACGGCCAGG-3′ R-5′-GATTCCATACCCAAGAAGGAAGG-3′.

### Immunofluorescence

For whole mount staining of tissues, yolk sacs or embryos were fixed overnight in 4% paraformaldehyde at 4 °C. Following fixation, tissues were washed extensively in PBS and skin was dissected from the dorsal region of embryos. Yolk sacs and skin were blocked for 1 h at room temperature in 1% bovine serum albumin, 0.3% Triton-X in PBS, followed by incubation with primary antibodies diluted in blocking solution overnight at 4 °C. Yolk sacs were stained with rat anti-CD31 (BD Pharmingen, 553370, 1:500) and rabbit anti-mouse LYVE-1 (Angiobio, 1:1000). Skins were stained with rat anti-CD31 and goat anti-Prox1 (R&D Systems, AF2727, 1:250). Tissues were washed extensively in PBS containing 0.1% Triton X-100 (PBS-T), then were incubated overnight at 4 °C with Alexa Fluor-conjugated secondary antibodies (Molecular Probes, Life Technologies) diluted in blocking solution. The following day, tissues were washed extensively in PBS-T and mounted in DAPI Fluoromount G (ProSciTech). Tissues were imaged using confocal microscopy as previously described^49^ using a Zeiss LSM 700 confocal microscope (Zeiss Laboratories). Images were compiled using ZEN lite 2012 (black edition) version 8.1. Adult mice were perfused with 4% PFA/PBS, brains were removed, postfixed for a further 16 hours, cryoprotected in 30% sucrose, and embedded in OCT medium. Sections (12 μm) were prepared using a Leica CM1900 cryostat. Blocking in PBS/0.1% Triton X-100/10% horse serum was performed for 30 minutes, followed by overnight incubation at 4 °C in the same solution containing diluted primary antibody. Sections were washed 3 times for 10 minutes in PBS and incubated in diluted secondary antibody at 4 °C for 6 hours. After washing 3 times for 10 minutes each in PBS, slides were mounted in Prolong Gold Antifade plus DAPI (Molecular Probes, Invitrogen) and imaged using a Nikon Eclipse Ti microscope and Nikon Digital Sight DS-Qi1 camera. Antibodies and their corresponding dilutions were: rabbit anti phospho-S6 Ribosomal Protein (Ser235/236) (Cell Signalling, #2211, 1:1000), rabbit anti phospho-S6 Ribosomal Protein (Ser240/244) (Cell Signalling, #5364, 1:1000), mouse anti-NeuN (Millipore, #MAB377, 1:100).

### Protein extraction and western blotting

Snap frozen embryos or brains for protein extraction were minced in extraction buffer (150 mM NaCl, 1% NP-40, 0.5% deoxycholate, 0.1%SDS, 50 mM Tris-HCl pH 7.5) and incubated at 4 °C for 30 minutes. Lysates were separated on Invitrogen Bolt precast 4–12% polyacrylamide gels and transferred to PVDF membrane before blotting. Antibodies and their corresponding dilutions were: rabbit anti phospho-S6 Ribosomal Protein (Ser235/236) (Cell Signalling, #2211, 1:1000) rabbit anti phospho-S6 Ribosomal Protein (Ser240/244) (Cell Signalling, #5364, 1:1000), rabbit anti phospho-p70 S6 Kinase (Thr389) (Cell Signalling, #9234, 1:1000), rabbit anti phospho-AKT (Ser473) (Cell Signalling, #4060, 1:2000), rabbit anti phospho-AKT (Thr308) (Cell Signalling, #13038, 1:750), rabbit anti p70 S6 Kinase (Cell Signalling, #9202, 1:500), rabbit anti S6 Ribosomal Protein (Cell Signalling, #2217, 1:750) and rabbit anti mTOR (Cell Signalling, #2983, 1:1000).

### Detection of DEPDC5 using parallel reaction monitoring (PRM) and selected ion monitoring (SIM) nano-LC-ESI-MS/MS

Protein lysates from heads of one 12.5 dpc *Depdc5* ^FS/+^ embryo and one 12.5dpc *Depdc5* ^+/+^ embryo were quantified using an EZQ protein assay (Life Technologies) and 2 µg of each sample was trypsin digested using the FASP method as previously described by^50^. LC-ESI-MS/MS was performed using an Ultimate 3000 nano-flow system (Dionex) coupled to an Impact II QTOF mass spectrometer (Bruker Daltonics) via an Advance CaptiveSpray source (Bruker Daltonics). 10 uL of each digested sample was loaded onto a trapping column (Acclaim PepMap100 C18, pore size 100 Å, particle size 3 µm, 75 µm × 2 cm length, Thermo Scientific) at 5 µL/min using 0.1% FA, 2% ACN in water for 10 min. Peptide separation was performed on a Acclaim PepMap RSLC column (C18, pore size 100 Å, particle size 2 µm, 75 µm × 50 cm length, Thermo Scientific) at 0.2 µL/min using a linear gradient of 5–45% ACN in 0.1% FA over 130 min.

PRM was performed on the QTOF using the following parameters for the peptide AVNGFLADLFTK; m/z 648.3533, collision energy of 26, and an isolation width of 4. Data was analysed using the open source software Skyline where it was compared to a DEPD5 spectral library acquired PeptideAtlas (http://www.peptideatlas.org/speclib/). The following Skyline settings were used; trypsin digestion, no missed trypsin cleavages, variable modifications of carbamidomethyl and oxidation, and mass error tolerance of 40 ppm.

Selected ion monitoring (SIM) nano-LC-ESI-MS/MS was performed using an Ultimate 3000 RSLC system (Thermo-Fisher Scientific, Waltham, USA) coupled to an Impact HD™ QTOF mass spectrometer (Bruker Daltonics, Bremen, Germany) via an Advance CaptiveSpray source (Bruker Daltonics). SIM is highly sensitive form of mass spectrometry as only peptides of interest are targeted for fragmentation and detection (based on their m/z) whilst all other peptides are excluded from data acquisition. 500 ng of the tryptic digests were pre-concentrated onto a C18 trapping column (Acclaim PepMap100 C18 75 μm × 20 mm, Thermo-Fisher Scientific) at a flow rate of 5 μL/min in 2% (v/v) ACN 0.1% (v/v) FA for 10 minutes. Peptide separation was performed using a 75 μm ID C18 column (Acclaim PepMap100 C18 75 μm × 50 cm, Thermo-Fisher Scientific) at a flow rate of 0.2 μL/minutes using a linear gradient from 5 to 45% B (A: 5% (v/v) ACN 0.1% (v/v) FA, B: 80% (v/v) ACN 0.1% (v/v) FA) over 70 minutes, followed by a 20 minute wash with 90% B, and a 20 minute equilibration with 5% A. A proteotypic peptide from DEPDC5 was targeted in the SIM analysis; TFIYGFYFYK (*m*/*z* 674.83) at a mass width of 0.1 Da with a collision induced dissociation energy of 33. The acquired spectra were analysed using the MaxQuant software (version 1.5.2.8) with the Andromeda search engine. The standard Bruker QTOF settings were used with a mass error tolerance of 40 ppm and the digestion enzyme specified as trypsin. The peptide and protein false discovery rates (FDR) was set to 1%. The dot-product (dotp) is a calculation for the normalized contrast angle, a comparison value between the peak areas (from the PRM acquisition) and the matching MS/MS peak intensities (from the spectral library).

### Mouse Embryonic Fibroblast isolation and immortalisation

Mouse embryonic fibroblasts were isolated from eviscerated 13.5 dpc trunks. Following tryptic digestion, cells were cultured on standard tissue culture plastic in DMEM supplemented with 10% FCS, 1% glutamax and 1% penicillin/streptomycin. MEFs were immortalised by serial passage. For starvation experiments MEFs were cultured in DMEM lacking amino acids (US Biologicals, # D9800-13) and without serum supplementation. Rapamycin (Cayman Chemicals) was used at a final concentration of 20 nM.

### Neurosphere growth and differentiation

Neurospheres were isolated from 13.5 dpc cortices. Cortices were stripped of meninges, minced with a scalpel blade and digested in papain (Worthington, #LK003178) supplemented with DNase (40 μg/mL, Worthington, #LS002139). Digestion was inhibited with a 1:1 mixture of BSA (Sigma) and ovomucoid (Worthington) in PBS and tissue triturated with fire polished glass pipettes. Single cells were cultured in neurosphere media (25% DMEM, 25% F12, 50% neurobasal, 2% B27, 10 ng/mL EGF, 20 ng/mL bFGF2). Subsequent passaging was performed with accutase (Sigma) by rinsing spheres in Ca/Mg free PBS, resuspending in accutase for 10 minutes and triturating with fire polished glass pipettes. Neurospheres were dissociated and differentiated for 3 days to a population of SOX3-positive neural progenitors for starvation experiments.

### Whole animal phenotyping

Four 12–18 month old male and female mice were sent for whole animal phenotyping to the Australian Phenomics Network. Histological analysis of 21 tissues and organs was conducted by a veterinary pathologist.

### Daily and nocturnal monitoring

Thirty minute daily observations were performed on 8 female and 8 male heterozygous mice from weaning to 12 weeks under standard husbandry conditions. Nocturnal monitoring was performed by recording with an infrared camera mounted above a perspex covered cage in which mice had free access to food and hydration gel. Cohorts consisted of eight heterozygous (four male, four female) and six control wildtype mice (three male, three female), aged 16–20 weeks. Each mouse was recorded for 12 hours.

### PTZ assay

Seizure susceptibility was measured by subcutaneous injection of pentylenetetrazole (PTZ) (120 mg/kg). 9 *Depdc5* ^+/+^ and 14 *Depdc5* ^FS/+^ 4–6 month old male mice were observed individually in clear cylinders for drug-induced seizure phenotypes culminating in tonic-clonic seizure. Other measures of seizure susceptibility including seizure duration and severity were not recorded due to ethical considerations.

### Statistical Analysis

The Lymphatic Vessel Analysis Protocol (LVAP)^51^, Image J plug-in was used to analyse lymphatic vessel branch points and vessel diameter. One image per embryo was used to analyse lymphatic vessel branch point. Three embryos across two different litters were analysed for each genotype (n = 3 per genotype, p = 0.04953). Two way comparisons were performed using Mann Whitney U tests for both parameters. The LVAP was also used to analyse lymphatic vessel diameter as previously described^51^. Briefly, a grid was applied to each image, and the lymphatic vessel diameter was measured at the point at which the horizontal and vertical grid lines intersected. A minimum of 30 measurements were recorded per image, (n = 3 per genotype, p < 0.0001)

Densitometric quantification was performed on three biological replicates on a single Western blot using a Biorad Chemidoc with Image Lab software. Three independent embryo samples were run for each genotype and two way comparisons were performed using unpaired two tailed Student’s t-tests. PTZ survival plots were compared using a Mantel-Cox test. Observed and expected numbers of embryos or offspring with pooled FS/+ and +/+ compared to FS/FS using a Chi-square test. NIS software was used to measure mean intensity of PS6 240/244 stained neurons by selecting and measuring the 5 brightest cells per field of view and subtracting a background non-stained measurement (n = 3 per genotype, 6 images taken per animal with at least 30 cells counted per animal). Mean intensity was compared using unpaired two-tailed Student’s t-test. NIS software was used to measure soma size in Nissl-stained sections (n = 3 of each genotype, 50 cells measured with the largest 30 used for statistical analysis (two-tailed Student’s t-test). All statistical tests were performed using GraphPad Prism 6 software.

## Electronic supplementary material

Supplementary Data
