# Supplementary material for: Knockout of the epilepsy gene Depdc5 in mice causes severe embryonic dysmorphology with hyperactivity of mTORC1 signalling
Source: Sci Rep. 2017 Oct 3;7:12618. doi: 10.1038/s41598-017-12574-2 (PMC5626732; doi:10.1038/s41598-017-12574-2)
Supplement: Supplementary file 1 — Supplementary Data [file 41598_2017_12574_MOESM1_ESM.pdf]

Supplementary Information

Knockout of the epilepsy gene Depdc5 in mice causes severe embryonic dysmorphology with hyperactivity of mTOR signalling

James Hughes[1,2*], Ruby Dawson[1,2*], Melinda Tea[3], Dale McAninch[1,2], Sandra Piltz[1,2], Dominique Jackson[1,2], Laura Stewart[1,2], Michael G. Ricos[4], Leanne Dibbens[4], Natasha Harvey[3], Paul Thomas[1,2,**]

[1]School of Biological Sciences, University of Adelaide, Adelaide, SA, AUS 5005

[2]Robinson Research Institute, University of Adelaide, Adelaide, SA, AUS 5005

[3]Centre for Cancer Biology, Division of Haematology, Adelaide, SA, AUS, 5000

[4]University of South Australia, Epilepsy Research Program, School of Pharmacy and Medical Sciences, Adelaide, SA, AUS 5000

Short Title: Role of Depdc5 in growth and development

* *These authors contributed equally to this work*

***** Corresponding author:***

Ph. +61883135009

Fax: +61883134362

Email: paul.thomas@adelaide.edu.au

a

Founder	Editing Agent	Genotype	Transmission (F0xWT)
1	C1	WT/-22	WT/-22
2	C1	WT/-4/-7	WT/-4, WT/-7
3	C1	WT/+6	NA
4	C1	WT/-1/+1	WT/-1, WT/+1
5	C2	WT/-3	NA
6	C2	WT/+5	NA
7	C2	WT/-3	NA
8	T1	-3/-12	NA
9	T1	-3/-18	NA
10	T1	-13/-3/-4	WT/-3, WT/-4
11	T1	-3/-12	NA
12	T1	-3/-3/-3	NA

b

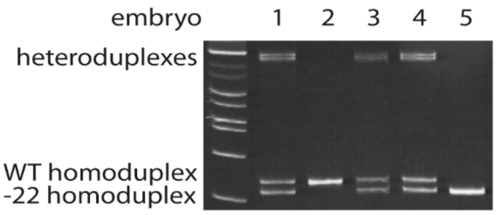

Supplementary Figure S1
Generation of *Depdc5* null mice.
(**a**) A total of 12 founders were generated with four selected for further breeding (#1, #2, #4, #10) to demonstrate transmission and establish frame shift lines. (**b**) Example polyacrylamide gel electrophoresis showing genotyping of 12.5dpc embryos from a +/-22 x +/-22 (derived from founder #1) showing that +/+ (embryo 2) FS/FS (embryo 5) and FS/+ (embryos 1, 3, 4) can be discriminated based on size. Note the presence of heteroduplexes with retarded electromobility in lanes 1, 3 and 4 indicative of heterozygosity.

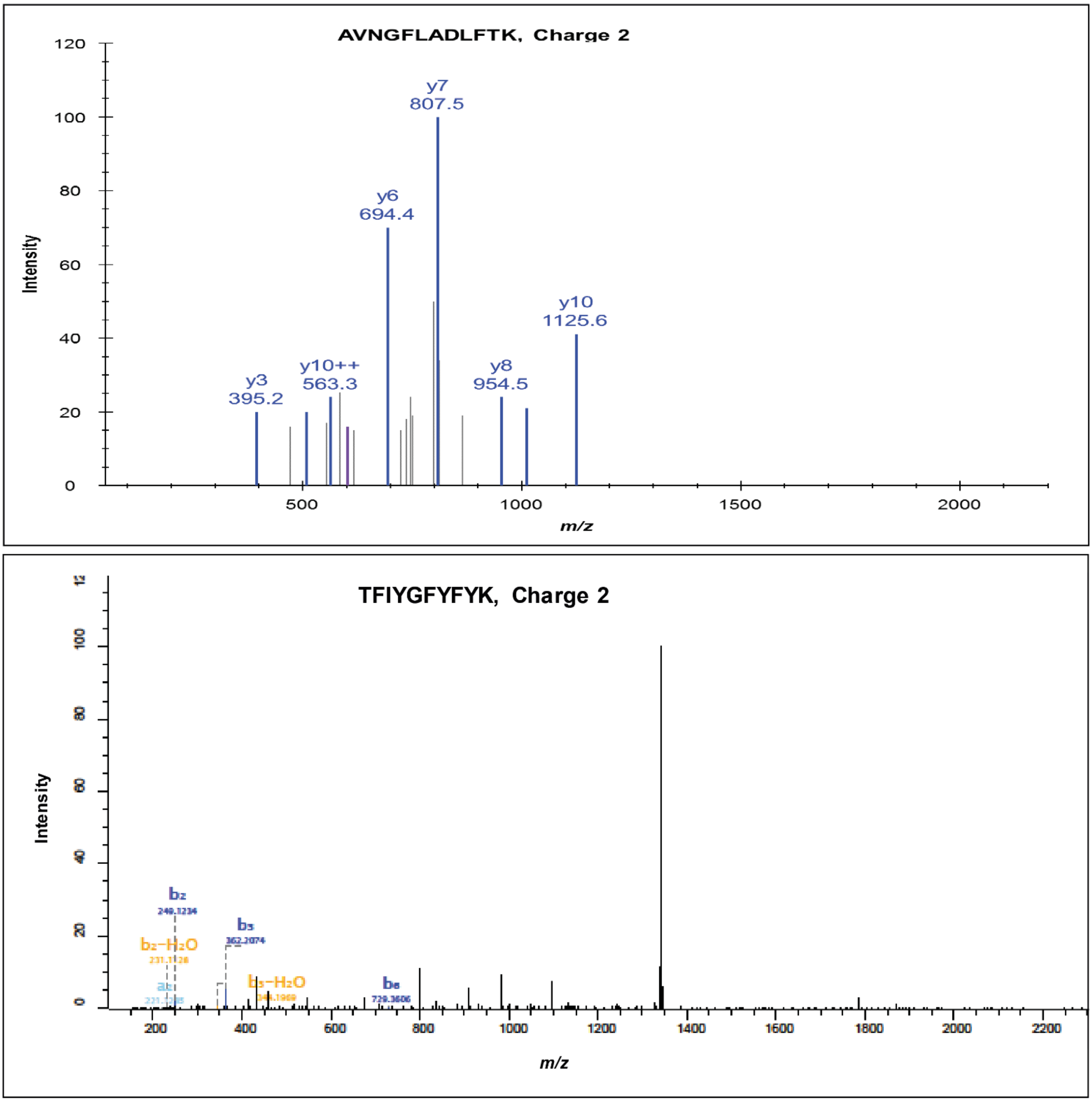

Supplementary Figure S2
Mass spectra of two DEPDC5 peptides. Mass spectra of AVNGFLADLFTK (m/z 648.35, +2) (top panel) and TFIYGFYFY (m/z 674.81, +2) (bottom panel) that were detected in brain lysate from a 13.5 dpc *Depdc5* $^{+/+}$ embryo.

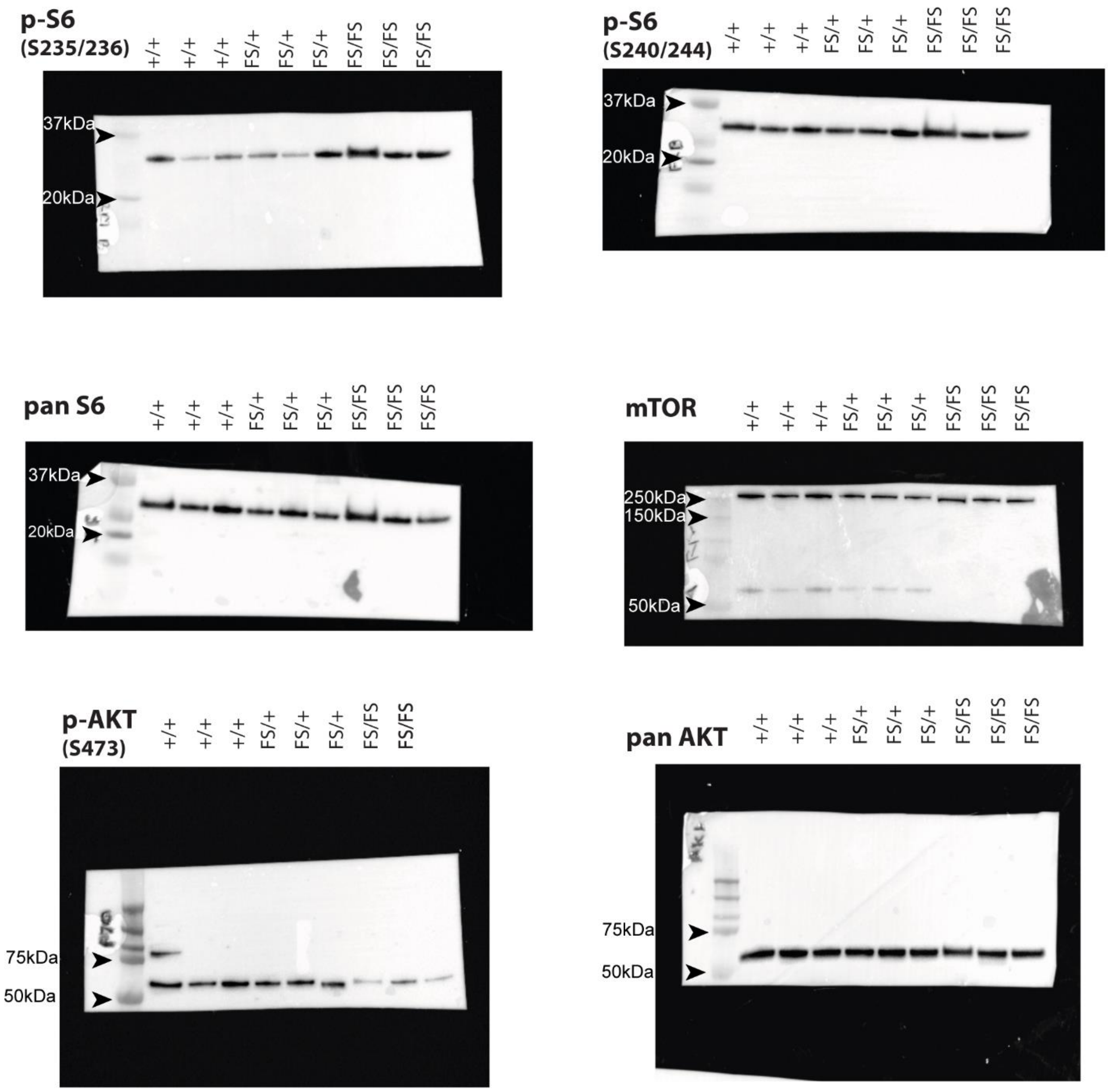

Supplementary Figure S3

Fulll length original uncontrasted Western blot images which are shown cropped in Figure 5. Marker sizes are labelled with arrows.

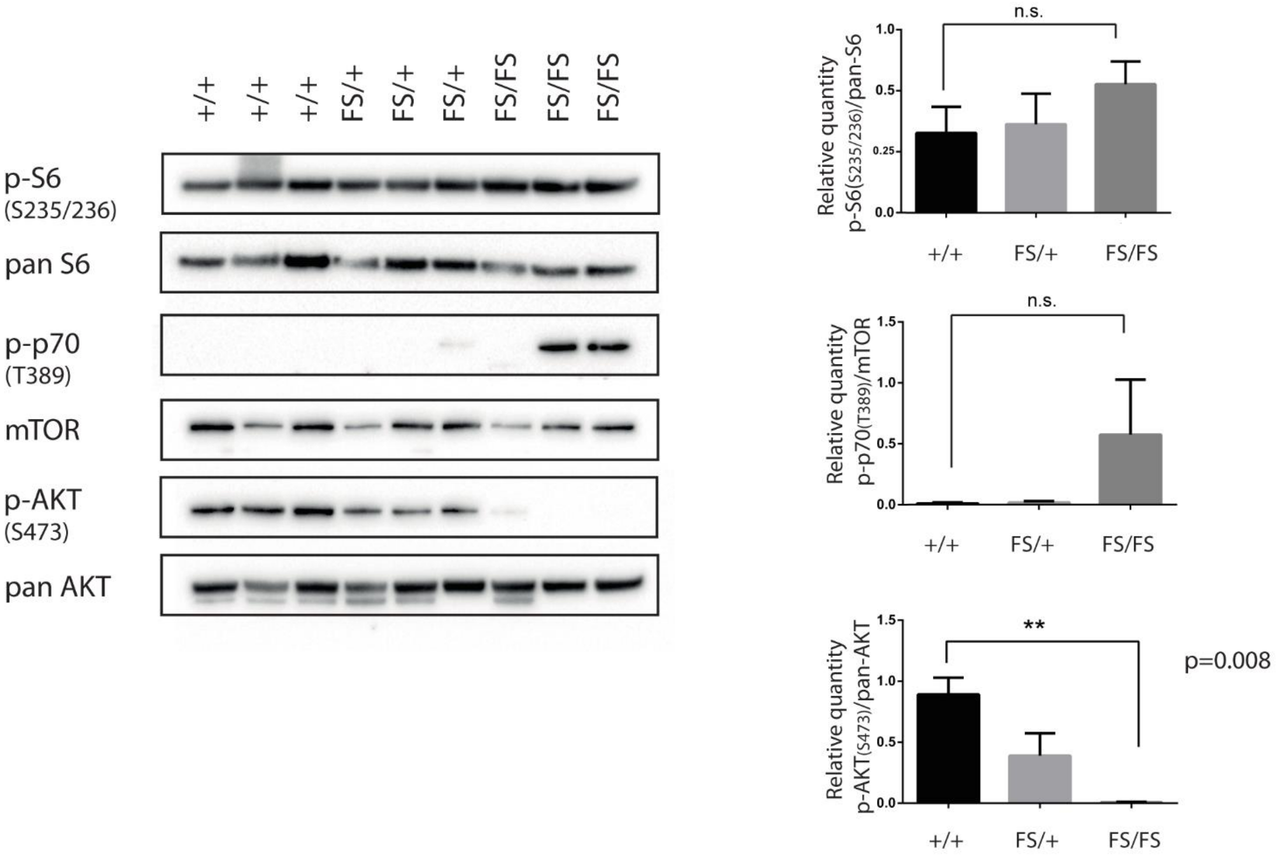

Supplementary Figure S4

mTORC1 signalling is upregulated in whole *Depdc5* $^{FS/FS}$ 9.5dpc embryo lysates. Three embryos from each genotype were used (n=3) and densitometric quantification was performed using unpaired two-tailed Student's t-tests, error bars represent SEM, **p=<0.01, n.s = not significant. Single seperate blots were used for each antibody using the same experimental samples and were processed in parallel. The images shown are cropped and full length original images are shown in Supplementary Fig. S5.

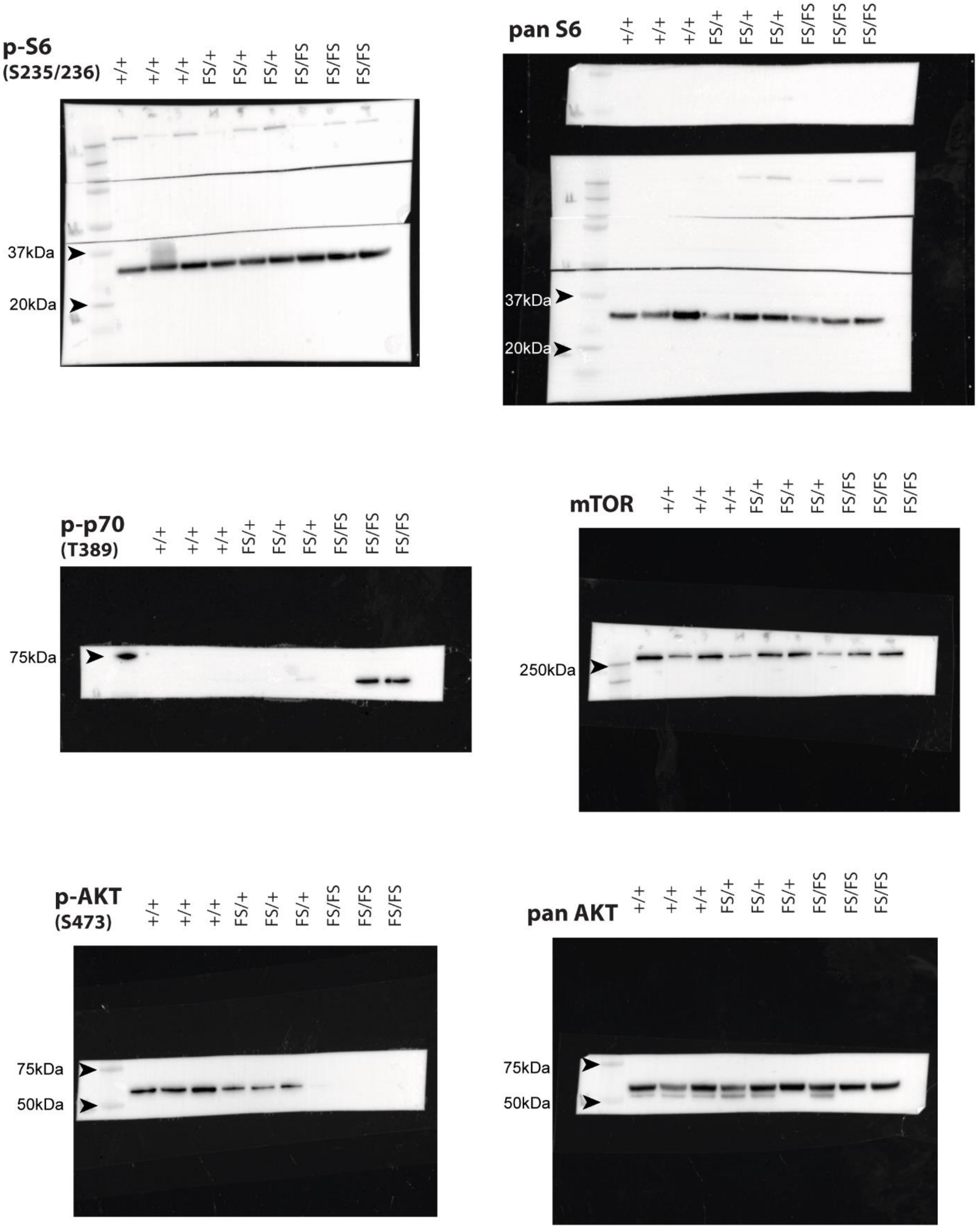

Supplementary Figure S5

Fulll length original uncontrasted Western blots which are shown cropped in Supplementary Figure S4. Marker sizes are labelled with arrows.

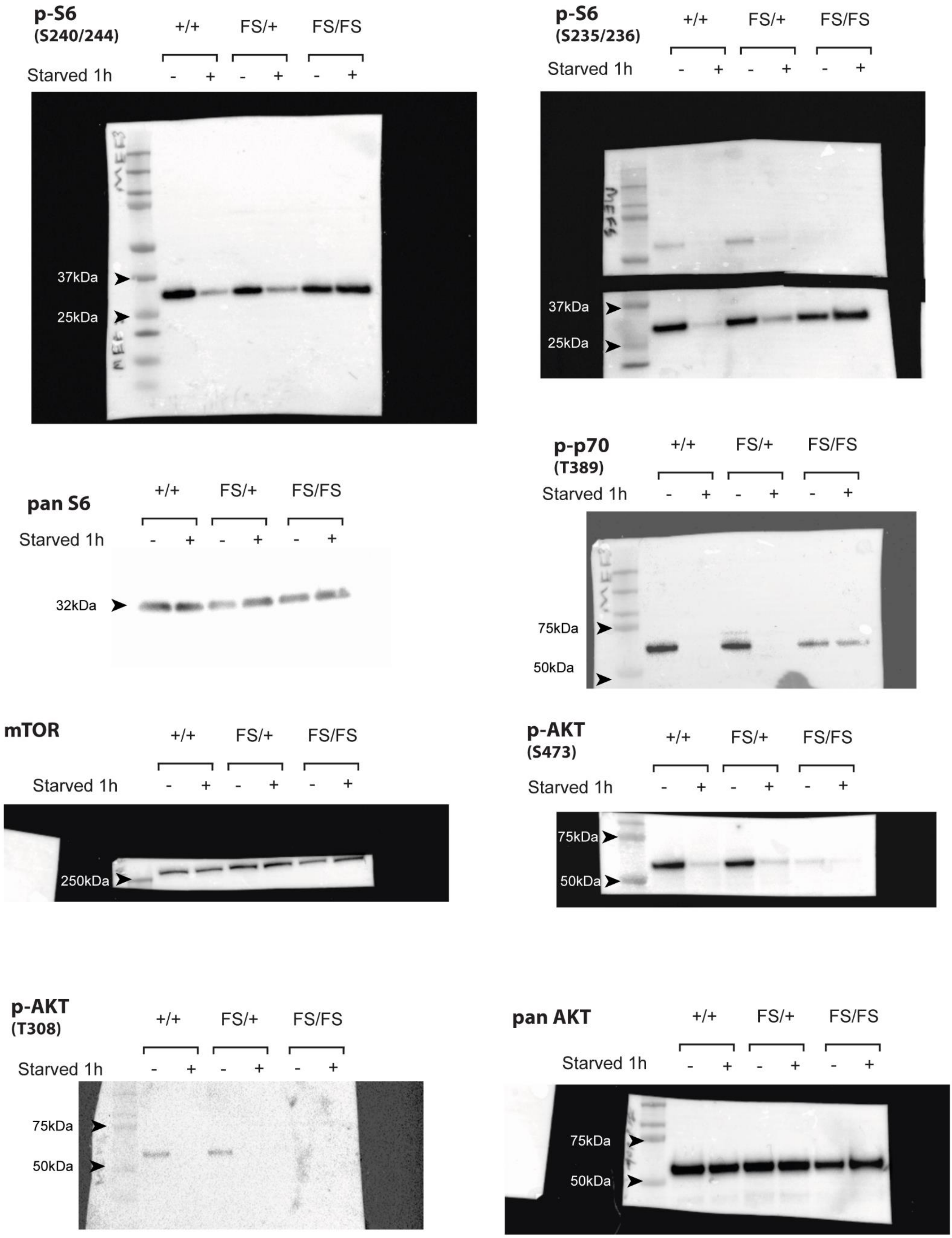

Supplementary Figure S6

Fulll length original uncontrasted Western blots which are shown cropped in Figure 6a. Marker sizes and predicted protein size (pan S6) are labelled with arrows.

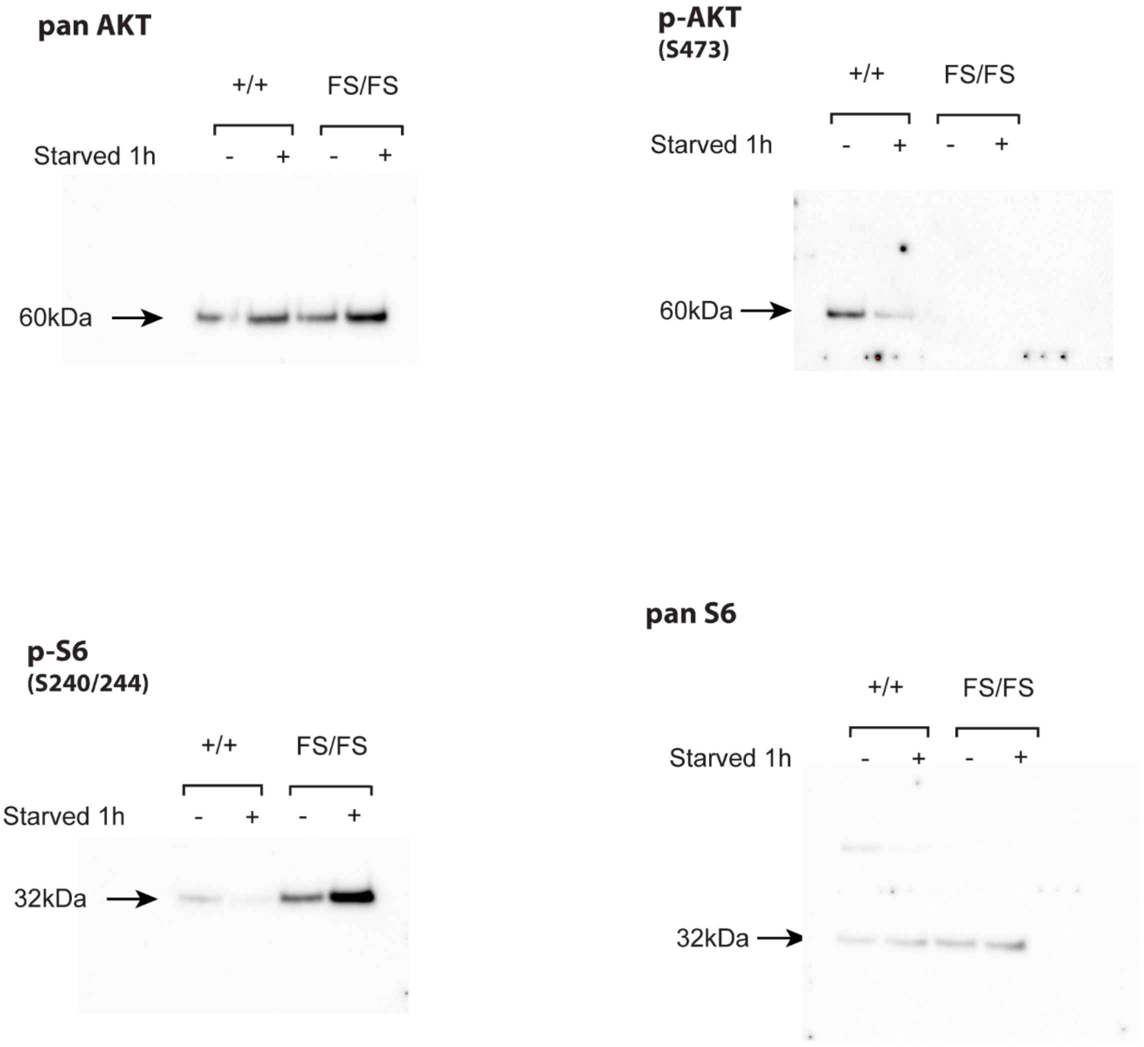

Supplementary Figure S7

Fulll length original uncontrasted Western blots which are shown cropped in Figure 6c. Predicted protein sizes are labelled with arrows.
